# Care of the Mouth and Teeth

**Published:** 1894-03

**Authors:** 


### EDITORIAL.

### Care of the Mouth and Teeth.

In the consideration of this subject, both in writing and discussion, the chief attention is usually given to the teeth and the immediate tissues, and that too after they have become diseased.

The matter of preventing disease does not usually receive the attention its importance demands.

The course of life and habit, that will secure in the highest possible degree a good state of health and prevent the occurrence of disease, is a subject that ought to be very thoroughly understood. The almost universal suggestion of those who speak or write upon this subject, to keep the teeth clean, is very good so far as it goes, indeed, this is an absolute requirement if the best health condition is to be maintained, but mere local attention, however thorough it may be, will come far short of securing the best results. Attention should be given to the secretions of the mouth, to the digestive organs, to the respiratory organs, and indeed to the condition of the entire system.

It is impossible to have a healthy mouth, with diseased digestive organs, or indeed with an impaired condition of any of the principal functions of the body; hence, general hygiene is a subject with which the dentist should be entirely familiar, but so rarely is a perfectly healthy mouth with a good set of teeth presented that the greater share of attention must be to those more or less impaired; and here attention to the general condition must be given as well, for restoration or even mitigation of disease in the mouth will depend very much upon the environment. Both local and general treatment will usually be required for the proper management of the diseases of the soft tissues of the mouth.

A very common fault with dentists is the failure to sufficiently emphasize to those they have in charge the importance of care, whether of cleanliness or general attention to health. A part once having been diseased, though the utmost may have been done for its reparation, is subsequently more liable to disease than before it was attacked. For instance, teeth that have been filled and are neglected are more subject to decay than before disease occurred ; there are, of course, some exceptions to this rule ; there may have been original defects in the teeth constituting vulnerable points for the attack of disease, but every one will recognize the fact that an attack of disease always irreparably weakens the organs, and it is rare that its original integrity can be restored. Under favorable circumstances repair in a large proportion of cases may be in a good degree effected. Not only should great care be bestowed upon repaired natural teeth, but assiduous and persistent attention should be given to all cases where artificial substitutes have been adopted, even the more simple varities of these, as, for example, a porcelain or gold crown must have intelligent and thorough care if it is to be kept in an acceptable condition ; and more care, if possible, should be given to artificial teeth supplied by bridges or by plates.

In the construction of artificial teeth, whether one or many, one of the main points to which attention should be given is such an arrangement as will facilitate the utmost cleanliness and freedom from irritants. This is, if possible, of more importance where a number of natural teeth remain in the mouth. The usual condition attaching to artificial teeth, whether on bridges or plates, is one of impurity and of offense, depending usually to a degree upon faulty construction and adaptation, and too often with carelessness on the part of the patient. Such a condition is an offense not only to the patient but to his friends and those with whom he comes in contact as well. Doubtless, many a time, patients suffer from indigestion and defective nutrition, the respiratory apparatus also becoming involved, by the neglected and filthy condition allowed to exist in connection with artificial teeth.

The proper construction of artificial dentures for securing the best results in these particulars should have more attention in the future than it has in the past.

---

In replying to an editorial note which appears in the February number of *The Pacific Coast Dentist*, we may say, when the Directory of State and Local Societies was first compiled it was supposed that the officers would, at least, take enough interest in those Societies to send notice of any change either in the election of officers or time of meeting; this, however, does not seem to be the case, as we find it necessary to write to some of the Corresponding Secretaries twice, and to a few even three times before receiving any reply. The pages of the Dental Register are always open for the publication of any notices in connection with the Societies, either State or Local, that may be sent in. We are much obliged for the notice of the change in the names of the officers connected with *The San Francisco Dental Society*, though not made known in the usual way. The correction will appear in the April number.

